# Physical activity counselling in people with suicidal ideation: a secondary analysis of a pilot study in Ugandan primary care settings

**DOI:** 10.11604/pamj.2024.48.160.39919

**Published:** 2024-08-07

**Authors:** Davy Vancampfort, James Mugisha, Simon Rosenbaum, Tine Van Damme

**Affiliations:** 1 KU Leuven Department of Rehabilitation Sciences, Leuven, Belgium,; 2 University Psychiatric Centre KU Leuven, Leuven-Kortenberg, Belgium,; 3 Department of Sociology and Social Administration, Kyambogo University, Kampala, Uganda,; 4 School of Psychiatry, University of New South Wales, Sydney, New South Wales, Australia

**Keywords:** Anxiety, depression, physical activity, suicide

## Abstract

**Introduction:**

primary care settings are ideal to implement suicide risk reduction initiatives in low-and middle-income countries. Health staff working in primary care settings are often over-burdened and under-resourced. Task-shifting through lifestyle counseling by lay health workers might be a relevant intervention. The aim of this secondary analysis from a pilot study exploring the efficacy of lay health worker (LHW)-led physical activity (PA) counselling for primary care patients with mental health problems (PCMH) was to investigate the efficacy of PA on reducing suicidal ideation.

**Methods:**

from 130 Ugandan PCMH screened in two centers, 8.5% (n=11) reported suicidal ideation. These 11 PCMH (9♀, median age= 52 years, interquartile range= 37 years) participated once weekly for 8 weeks in group PA counselling based on the mental contrasting and implementation of intentions framework. All participants completed the Patient Health Questionnaire-9 (PHQ-9), Generalized Anxiety Disorder-7 (GAD-7), and the Simple Physical Activity Questionnaire (SIMPAQ) pre- and immediately post-intervention.

**Results:**

in PCMH with suicidal ideation (PHQ-9 item 9≥1) the prevalence of suicidal ideation dropped to 9% post-intervention, i.e. only one patient reported suicidal ideation post-intervention. Following the intervention, significant (P<0.05) increases in walking, exercising and incidental PA (SIMPAQ) levels, and reductions in depressive and anxiety symptoms were observed.

**Conclusion:**

our data demonstrate that LHW-led PA counselling might be promising intervention in reducing suicidal ideation in primary care patients in low-resourced settings. Randomized controlled trials are warranted to confirm these beneficial findings.

## Introduction

Suicide is among the leading global causes of death, with over 75% of all suicides occurring in low-and middle-income countries (LMICs) [1]. A study in five LMICs demonstrated that 10.3% of people presenting at primary care facilities reported suicidal ideation within the past year, and 2.2% reported attempting suicide in the same period [2]. While in LMICs suicidal ideation ranges from 3.5 to 11.1% in community samples, this increases up to 14.8% in those attending primary care facilities [2]. Primary care settings are therefore ideal environments to implement suicide risk reduction initiatives. However, while in LMICs, such as Uganda, most primary care providers are knowledgeable about suicide and associated risk factors, they report challenges in assessing and managing individuals with suicide risk [3]. For example, a recent study in 7,958 health facilities across seven LMICs demonstrated that amitriptyline, an antidepressant classified as essential by the World Health Organization (WHO) [4], was available in only 8% of the primary care settings on the day of assessment [5]. Therefore, non-pharmacological interventions to reduce suicidal ideation among primary care patients with mental health problems (PCMH) are essential. However, a key gap is the lack of integration of psychotherapeutic interventions as a part of accessible evidence-based primary care [6]. One of the contributing factors is that most primary care centers in LMICs have very few trained mental health specialists and an overburdened workforce [7] resulting in psychological care often being delivered via task-shifting (i.e., having providers without specialized mental health training delivering mental health interventions) [8].

Recently, the role of lifestyle psychiatry has been acknowledged as a promising non-pharmacological strategy for reducing the mental health burden in LMICs, in particular when delivered via lay health workers [9,10]. Physical activity is a low-cost lifestyle intervention which demonstrated to be promising in reducing suicidal risk [11]. However, the few existing trials were executed in high-income countries and real-world interventions in low-income countries are lacking. Exploring the effectiveness and efficacy in real-world interventions and those delivered by non-experts, but supervised by experts, is important, particularly because drop-out rates from physical activity interventions are as high as 20 to 30% in people experiencing mental health problems high [12-15]. Therefore, in recent years, several calls were made to explore novel, innovative and culturally-sensitive approaches to reduce the burden of poor mental health via lifestyle interventions [10,16]. One strategy which has been applied successfully via task-shifting by lay health workers is the mental contrasting with implementation of intentions methodology [17]. Mental contrasting entails an expectancy-based form of goal-setting whereby after specifying a goal (e.g., to become more physically active) individuals denominate and imagine the most beneficial outcome of successfully changing their behavior (e.g., feeling more fit or energetic), followed by denominating and imagining the most salient obstacle that prevents them from realizing their goal (e.g., experienced fatigue) [17]. Implementation of intentions involves “if-then” plans that specify when, where, and how the goal intention should be implemented (e.g., “If I feel too tired for a walk, then I will call my support partner to join me.”) [18].

In a pilot study [19], we demonstrated that 8-weeks of weekly physical activity counseling based on the principles of mental contrasting and implementation of intentions significantly (P<0.001) increased walking, exercising and incidental physical activity levels, and reduced depressive and anxiety symptoms in PCMH. The aim of this secondary analysis of our pilot study was to explore the efficacy in reducing levels of suicidal ideation in those who presented at the start with suicidal ideation. A secondary aim of the current study was to explore whether any changes in suicidal ideation were accompanied by changes in depression, anxiety, and physical activity levels. We hypothesize that physical activity counselling delivered via lay health workers, under supervision of a psychiatrist clinical officer, will reduce suicidal ideation and is accompanied by reductions in anxiety and depression and increases in physical activity levels.

## Methods

**Study design:** this is a secondary analysis of a pre-test/post-test study without a control group [19].

**Study setting:** this study that took place in two health centers in poor communities in Mpigi district in Central Uganda.

### Study population

Patients attending the primary care center were eligible for the original pilot study if they (a) were aged 18 or older years, (b) met criteria for at least mild depressive symptoms (Patient Health Questionnaire-9 score of 5 or higher) [20], and / or anxiety symptoms (Generalized Anxiety Disorder - 7 score of 5 or higher) [21], (c) were not complying with the internationally recommended target of 150 minutes per week of moderate to vigorous physical activity as assessed with the Physical Activity Vital Sign method [22], and (d) had no risks for physical activity counseling as assessed with the Physical Activity Readiness Questionnaire [23]. In total 130 of 139 PCMH [median (interquartile range) age=47.0 (22.0); 73.1% (n=95) female] agreed to participate in the pre-intervention screening. The nine potential participants who declined were all not interested due to practical, professional reasons (e.g., too busy) and their reasons for decline were not related to clinical or health-related reasons. Of the 130 interested patients, 53 reported either mild symptoms of depression and/or anxiety and were physically inactive and therefore eligible. None had contra-indications for being physically active. Four participants dropped out and were lost for follow-up. Forty-nine patients completed the trial and of these 49, eleven presented with suicidal ideation at baseline. In agreement with recent Ugandan studies [24,25], suicidal ideation was in this secondary analysis considered present when participants scored 1 or higher on item 9 of the Patient Health Questionnaire-9 (PHQ-9) [20].

**Study sampling:** for one month, all primary care patients visiting one of the two health centers irrespective of the reason for visiting the health center and who agreed were screened by a psychiatrist clinical officer. The health centers were purposively selected by the local health district office as having a larger number of people with mental health issues as compared to other health centers. One health center was located in a relatively busy trading center while the other one was in a rural area.

### Study intervention

The study leaders first approached the local leader of the district health system who advised on the health facilities where this study was to be conducted. The health centers chosen in this study were at the level of Health Centre III (based on the Uganda Ministry of Health Administrative structure). The lowest administrative level in the health sector in Uganda is the village health team and this is equivalent to Health Centre I. These village health teams are normally at the center of most community-based health interventions [26]. Village health team members are unsalaried community health workers recommended by their own communities and with basic health training which last about 5 to 7 days [27]. They are normally at least 18 years old, should regularly reside in the village, and literate in the local language [28]. Selection criteria were being physically active (self-reported), and a community health champion (recognized by the village health team and local staff of the health centers), trust-worth and having experience in providing lay counselling. Selection was done by the staff of the involved health centers who knew the village health teams and local communities well.

The selected lay health workers received a one-day training in motivational interviewing [29] and in adopting the mental contrasting with implementation of intentions framework [17,18]. Within this framework [17,18] PCMH were facilitated by the lay health workers to consider and elaborate upon the most positive outcome they associated with achieving their daily physical activity goals. Elaboration included visualizing the events or experiences associated with this outcome considering the local context in the farming community. Following this, lay health workers requested PCMH to consider and elaborate upon important obstacles that could potentially impede their ability to achieve this daily physical activity goal. Prompting questions included, ‘Why would this make it hard for you to achieve your goal?’ and ‘When and where would this occur?’. Special attention was given by lay health workers, support partners and PCMH to the local context. PCMH were then asked to think of an action that would overcome the obstacle and complete the following sentence regarding each specified obstacle: ‘If I (obstacle), then I will (action)´. Participants were encouraged to repeat their if-then plans aloud and endeavor to implement these daily in the coming week. In formulating intentions and developing an action plan, the lay health workers paid special attention to the importance of social support. Social support partners were able to attend the sessions. Village health team members were regular monitored by the psychiatrist clinical officer while review meetings were organized every two weeks. The project team also supported activities regularly as they visited patients at village level and provided information, education and communication materials. For project sustainability, village health team members were encouraged to form groups for income generation and trained in entrepreneurship skills. More details of the intervention are described elsewhere [19].

### Study variables

**Socio-demographical and clinical variables:** patients were asked whether they had a paid job / were employed (yes versus no). Age, gender, smoking (yes versus no, and if yes how many cigarettes per day), and the presence of chronic somatic comorbidities (yes versus no, with conditions lasting longer than 3 months defined as chronic) were self-reported and, when possible, confirmed via the medical files. Multimorbidity was considered being present if two or more chronic somatic comorbidities were reported. For calculating the body mass index (BMI), weight was measured in light clothing to the nearest 0.1 kg using a SECA beam balance scale, and height to the nearest 0.1 cm using a wall-mounted stadiometer.

### Patient Health Questionnaire -9 (PHQ-9) [20]

Patients completed the Luganda version of the PHQ-9 [20] pre- and post-intervention. The nine items are based directly on the nine diagnostic criteria for major depressive disorder in the DSM-IV (American Psychiatric Association, 1994). For each item, the individual is asked to rate the severity of his or her symptoms over the past two weeks, scored on a Likert scale with symptoms rated as 0 (not at all), 1 (several days), 2 (more than half the days) and 3 (nearly every day). Higher scores indicate more severe symptoms of depression. Suicidal ideation was considered present when the participants scored 1 or higher on item 9 evaluating suicidal ideation in the past two weeks. The PHQ-9 has a good internal consistency reliability, test-retest reliability, and construct validity and has been used previously in people with mental health problems in Uganda [30,31]. The Cronbach´s alpha for the PHQ-9 in the pilot study was 0.74 [19].

### Generalized Anxiety Disorder -7 (GAD-7) [21]

Patients completed the Luganda version of the GAD-7 pre- and post-intervention. The GAD-7 is a seven-item instrument that is used to assess the severity of generalized anxiety disorder (GAD). For each item, the individual is asked to rate the severity of his or her symptoms over the past two weeks, by providing a score on a Likert scale with symptoms rated as 0 (not at all), 1 (several days), 2 (more than half the days) and 3 (nearly every day). Higher scores indicate more severe symptoms of generalized anxiety. The GAD-7 has been used previously in people with mental health problems in Uganda [32]. The Cronbach´s alpha for the GAD-7 in the pilot study was 0.80 [19].

### Simple Physical Activity Questionnaire (SIMPAQ) [33]

Physical activity was assessed pre- and post-intervention in patients and support partners with the Luganda version of the SIMPAQ [33]. The SIMPAQ [33] is a 5-item clinical tool to assess physical activity among populations at high risk for sedentary behavior. It uses an interview format to estimate time spent in bed (min/day), time spent sedentary during waking hours (min/day), time spent napping (min/day), time spent walking (min/day), time spent in structured exercise (min/day), and time spent in incidental or non-structured physical activity (min/day) during the past week. The sum of the hours recorded in the SIMPAQ items should add to approximately 24-hours, providing interviewers with an opportunity to clarify if participants significantly under or over-reported (e.g. total of <18 hours or >30 hours accounted for). Previous research in Uganda demonstrated the questionnaire is reliable [34], while validity has been demonstrated in a 23-country validation study [35]. In this study, we used time spent walking (min/day), time spent in structured exercise (min/day), and time spent in incidental or non-structured physical activity (min/day) during the past week. Digging was considered a structured activity of moderate to vigorous intensity and therefore presented as an example under structured exercise in this farmer community.

**Study procedure:** all eleven participants with suicidal ideation were assessed pre- and immediately post-intervention.

**Statistical analyses:** data were tested for normality using the Shapiro-Wilks test and found not to be normally distributed. Changes in outcome measures were evaluated using Wilcoxon signed-rank tests (P< 0.05). SPSS version 28.0 (SPSS Inc., Chicago) was used for all data analyses.

**Ethical considerations:** written informed consent was obtained from all these participants, with illiterate participants providing consent via a fingerprint. The pilot study was approved by the ethical committee of Mengo Hospital.

## Results

### Participants

Of the 130 PCMH screened at baseline, eleven reported suicidal ideations (prevalence = 8.5%). These 11 PCMH [median (interquartile range, IQR) age=52.0 (37.0); 9 women] were included in this secondary analysis study. Six participants reported to be a farmer, one a tailor and four were not working or retired. The median body mass index was 19.7 (IQR=3.6). Two participants smoked and both reported smoking 2 cigarettes per day. One patient reported chronic asthma, one chronic cardiovascular symptoms, one chronic low back pain and two chronic joint pain. One patient suffered longer than 3 months from abdominal pain. No patient reported multi-morbidity, i.e. two or more chronic conditions, indicating that 6 out of 11 patients reported having a chronic physical condition.

*Changes in suicidal ideation, depression, anxiety and physical activity levels following the 8-week lay health workers- led physical activity counselling in PCMH*

Between baseline and 8 weeks, the prevalence of suicidal ideation reduced from 100 to 9.1% (P=0.002). Only one participant reported suicidal ideation post-intervention. The score on item 9 for suicidal ideation on the PHQ-9 increased in this patient from 1 to 3. Other changes in time of the clinical characteristics in PCMH are presented in Table 1.

**Table 1:** changes in clinical variables in primary care patients with suicidal ideation (n=11) following 8 weeks of lay health workers led physical activity counselling

Variables	Pre-test Median (interquartile range) or %	Post-test Median (interquartile range) or %	P
Suicidal ideation (number, prevalence, %)	11/11, 100%	1/11, 9.1%	0.002*
PHQ-9 total score	11.0 (8.0)	3.0 (3.0)	0.003*
GAD-7 total score	8.0 (5.0)	2.0 (5.0)	0.006*
SIMPAQ walking (min/day)	4.0 (13.0)	39.0 (34.0)	0.004*
SIMPAQ exercise (min/day)	0.0 (0.0)	97.0 (120.0)	0.005*
SIMPAQ incidental (min/day)	20.0 (20.0)	180.0 (60.0)	0.005*

* Significant when P<0.05. GAD-7 = Generalized Anxiety Disorder - 7, PHQ -9= Patient Health Questionnaire, SIMPAQ=Simple Physical Activity Questionnaire.

## Discussion

To the best of our knowledge, the current pilot study is the first to demonstrate that in a poor farming community, 8-weeks of lay health worker-led physical activity counselling based on mental contrasting with implementation of intentions principles [17], is associated with a reduction in suicidal ideation in the majority of PCMH reporting suicidal risk. Our findings are clinically relevant since the prevalence of suicidal ideation in the screened PMCH is roughly one in ten, which is in line with the 5.0-14.8% range observed in those attending primary care facilities in other LMICs [2]. Second, the findings are clinically relevant since existing key staff members in primary care centers experience challenges in assessing and managing individuals with suicide risk [7]. Our data suggest that task-shifting and involving lay health workers who could focus on lifestyle counseling might be a promising component of treatment. However, lifestyle counseling is not a stand-alone treatment and should be offered within a multidisciplinary approach [9], in particular knowing that in one of the 11 participants suicidal ideation worsened. Therefore, supervision by an expert such as a psychiatrist clinical officer is essential.

A secondary finding was that in the eleven patients, levels of depression and anxiety also reduced, while levels of physical activity increased. One of the potential underlying mechanisms for the reduced suicidal ideation therefore might be the observed reduction in depressive and anxiety feelings. Both of which are known risk factors for suicide in Uganda [36,37], and likely reduced in response to participants´ being more physically active. There are several neurobiological and psychosocial pathways that could clarify the observed reduction in suicidal ideation and mental health symptoms associated with being more physically active. For example, neurobiological changes such as an increased cerebral blood flow and changes in peripheral biomarkers such as an increase in circulating neurotrophic growth factors, and anti-inflammatory markers have been reported before [38]. From a psychosocial perspective physical activity provides people with an opportunity for social interaction (relatedness) and independence (autonomy) when recovering, and mastery in the physical domain (increased self-efficacy and perceived competence) [39]. For the latter, the potential efficacy of mental contrasting with implementation of intentions principles is of particular of interest. The methodology exhibits several advantages as an augmentation to usual care in those at risk for suicidal behavior in primary care settings. Not only does it increase commitment to cope with mental health problems, but it also enables people to identify the significance of daily life obstacles that act as barriers. Moreover, it motivates patients to overcome the identified obstacles [17]. The methodology is simple to use, brief, and an engaging exercise which does not require lengthy training. Our study suggests that it could also be a valuable approach in a resource and time-limited primary health care context and can be delivered by lay health workers in poor communities as a vital add-on strategy to increase access to mental health care [40].

### Limitations and future research

The findings of the present study should be interpreted with caution due to some methodological limitations. First, although suicidal ideation reported via item 9 of the PHQ-9 is a robust predictor of suicide attempts and deaths regardless of age [41], it should be noted that the PHQ-9 was designed to screen for depression and assess its severity, not to assess risk for suicide. Second, the one-group quasi-experimental research design with a modest sample size limits the validity and generalizability of the current findings. Third, physical activity was only measured with a self-report questionnaire, which is prone to both systematic and random errors [42]. Fourth, no long-term follow-up was done after the counselling cessation. Fifth, we did not have information on concurrent psychotropic medication use and adherence. Randomized controlled studies using longer-term follow-up are therefore recommended before concluding that physical activity counselling led by lay health workers and using mental contrasting with implementation of intentions principles is an efficacious intervention for reducing suicidal ideation in low-resourced environments. Lastly, research is needed to investigate in more detail the cost-effectiveness of interventions led by lay health workers versus by health professionals in these challenging environments.

## Conclusion

Despite the reported limitations, this secondary analysis demonstrates that an 8-week physical activity counseling based on the principles of mental contrasting with implementation of intentions principles [17] is associated with lower suicidal ideation for PCMH. Reductions in suicidal ideation are accompanied with reductions in depressive and anxiety symptoms and higher levels of physical activity.

### What is known about this topic

- *Suicide is among the leading global causes of death, with over 75% of all suicides occurring in low-and middle-income countries;*
- *Most primary care providers in low- and middle-income countries are knowledgeable about suicide and associated risk factors, but they report challenges in assessing and managing suicide risk;*
- *Physical activity is a low-cost lifestyle intervention which demonstrated to be promising in reducing suicidal risk in some high-income countries*.

### What this study adds

- *Lay health workers-led physical activity counselling reduces suicidal ideation in primary care patients in low-resourced settings;*
- *Reduced suicidal ideation is accompanied by reduced levels of depression and anxiety, while levels of physical activity increased;*
- *The mental contrasting with implementation of intentions methodology might be a promising strategy to support primary care patients in low-resourced settings in becoming more physically active*.
